# Genetic characterization of H5N1 influenza viruses isolated from chickens in Indonesia in 2010

**DOI:** 10.1007/s11262-012-0722-0

**Published:** 2012-02-10

**Authors:** Chairul A. Nidom, Shinya Yamada, Reviany V. Nidom, Kadek Rahmawati, Muhamad Y. Alamudi, Kholik, Setyarina Indrasari, Ratnani S. Hayati, Kiyoko Iwatsuki Horimoto, Yoshihiro Kawaoka

**Affiliations:** 1 Avian Influenza-zoonosis Research Center, Airlangga University, East Java, Surabaya 60115 Indonesia; 2 Influenza Laboratory-Institute Tropical Disease, Airlangga University, East Java, Surabaya 60115 Indonesia; 3 Faculty of Veterinary Medicine, Airlangga University, East Java, Surabaya 60115 Indonesia; 4 Division of Virology, Department of Microbiology and Immunology, Institute of Medical Science, University of Tokyo, Shirokanedai, Minato-ku, Tokyo, 108-8639 Japan; 5 International Research Center for Infectious Diseases, Institute of Medical Science, University of Tokyo, Shirokanedai, Minato-ku, Tokyo, 108-8639 Japan; 6 Core Research for Evolutional Science and Technology, Japan Science and Technology Agency, Saitama, 332-0012 Japan; 7 Department of Pathological Sciences, School of Veterinary Medicine, University of Wisconsin-Madison, Madison, WI 53706 USA

**Keywords:** H5N1 influenza virus, Indonesia, Phylogenic analysis, Genetic characterization

## Abstract

Since 2003, highly pathogenic H5N1 avian influenza viruses have caused outbreaks among poultry in Indonesia every year, producing the highest number of human victims worldwide. However, little is known about the H5N1 influenza viruses that have been circulating there in recent years. We therefore conducted surveillance studies and isolated eight H5N1 viruses from chickens. Phylogenic analysis of their hemagglutinin and neuraminidase genes revealed that all eight viruses belonged to clade 2.1.3. However, on the basis of nucleotide differences, these viruses could be divided into two groups. Other viruses genetically closely related to these two groups of viruses were all Indonesian isolates, suggesting that these new isolates have been evolving within Indonesia. Among these viruses, two distinct viruses circulated in the Kalimantan islands during the same season in 2010. Our data reveal the continued evolution of H5N1 viruses in Indonesia.

## Introduction

Highly pathogenic H5N1 avian influenza A viruses have spread among domestic poultry in Asian, European, and African countries since they emerged among geese in the People’s Republic of China in 1996 [1]. Although most of these countries have successfully controlled or eradicated the disease by culling poultry populations, H5N1 influenza remains enzootic in Indonesia, Egypt, and China. In Indonesia, the first outbreaks of H5N1 influenza were reported in November 2003, and now the viruses has spread to 32 out of the 33 Indonesian provinces, affecting both intensively farmed birds as well as backyard chickens [2]. In Indonesia, 182 cases of human infection have been reported with 150 deaths [3]. This represents the highest case number and case fatality rate (>80%) of all affected countries [3]. As part of its virus control program, Indonesia introduced a vaccination campaign in August 2004. Vaccine was given to various species including layer chickens, broilers, backyard chicken, ducks, and quails. The policy to control and prevent avian influenza in Indonesia needed to be reviewed and promptly improved. However, information on the H5N1 influenza viruses circulating in Indonesia is not available in the publically accessible databases. Only five Indonesian isolates have been registered in such databases since 2009.

To understand the current situation regarding H5N1 influenza viruses circulation among chickens in Indonesia, we conducted surveillance studies throughout 2010. Here, we report the isolation and molecular characterization of H5N1 influenza viruses isolated from chickens in Indonesia in 2010.

## Materials and methods

### Sample collection

A total of 1,607 tracheal and cloacal swab samples were collected from healthy chickens at wet markets and chicken farms in Java, Kalimantan, Sumatra islands between April and December 2010. Samples were suspended in transport medium containing M199 and transported to the diagnostic laboratory in a refrigerated container. Samples were inoculated into 10-day-old embryonated eggs, and their allantoic fluids were tested for hemagglutinating activity as described elsewhere [4]. Hemagglutination-positive samples were subjected to sequencing analysis. All experiments with infectious H5N1 virus were performed in a biosafety level-3 containment laboratory.

### Sequence analysis

We extracted viral RNAs from virus-positive allantoic fluid by using ISOGEN (Nippon Gene, Tokyo, Japan) according to the manufacturer’s instructions. Extracted RNAs were reverse transcribed with SuperScript^TM^ III reverse transcriptase (Invitrogen, Carlsbad, CA) and an oligonucleotide complementary to the 12-nucleotide sequence at the 3′ end of the viral RNA and were then amplified by using PCR with Phusion (New England Biolabs, Ipswich, MA) and primers specific for each segment of H5N1 influenza virus. The PCR products were loaded on an agarose gel, purified with a Minelute Gel Extraction Kit (Qiagen, Hilden, Germany) and then sequenced. Sequences of primers are available upon request. The sequences obtained in this study are available from Genbank, accession nos. AB685448–AB685463.

### Phylogenetic analysis

To understand the interrelationship of H5N1 viruses isolated from chickens in Indonesia, we phylogenetically analyzed the viruses we isolated in this study, together with publicly available sequence data. We used sequence data of viruses that belonged to clades 2.1.1, 2.1.2, 2.1.3, and IDN/6/05-like and were isolated in Indonesia. All sequences were assembled and edited by using BioEdit 7 software [5] and a neighbor-joining (NJ) tree was constructed by using ClustalW. Estimates of the phylogenies were calculated by performing 100 NJ bootstrap replicates.

## Results and discussion

**Fig. 1:**
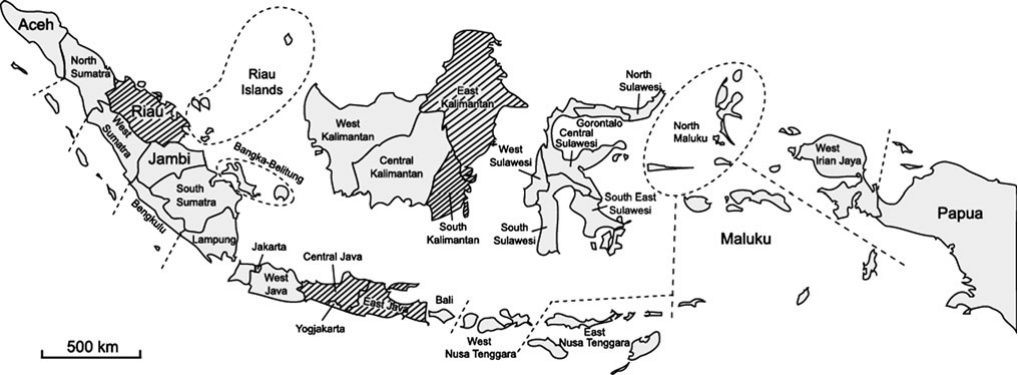
Provinces in Indonesia (*gray shading*) where surveillance for influenza A (H5N1) viruses in chickens was conducted in 2010

Of the 1,607 tracheal and cloacal swabs collected from chickens in Indonesia in 2010, eight influenza A viruses were isolated (0.50%) (Fig. 1; Table 1). In April, two viruses were isolated from samples collected in East Kalimantan. In May and June, one virus each was isolated from samples in East Java and East Kalimantan, respectively. In July and August, four viruses were isolated from samples collected in South Kalimantan, East Kalimantan, Sumatra, and Central Java (Fig. 1; Table 1).

**Table 1:** Influenza viruses isolated from chicken in our surveillance programs in Indonesia in 2010

Sampling season	Province	District	No. of samples	Virus isolation rate
April	East Kalimantan	Balikpapan, Samarinda,	279	2 (0.72%)
May	East Java	Kediri, Blitar, Surabaya	225	1 (0.44%)
June	East Kalimantan	Balikpapan	126	1 (0.79%)
July	East Kalimantan	Balikpapan	48	1 (2.08%)
	South Kalimantan	Banjarmasin	106	1 (0.94%)
	Riau (Sumatra)	Pekanbaru	207	1 (0.48%)
August	Central Java	Semarang	156	1 (0.64%)
November	East Java	Surabaya	178	0 (0.00%)
December	East Java	Surabaya	282	0 (0.00%)
		Total	1,607	8 (0.50%)

**Fig. 2:**
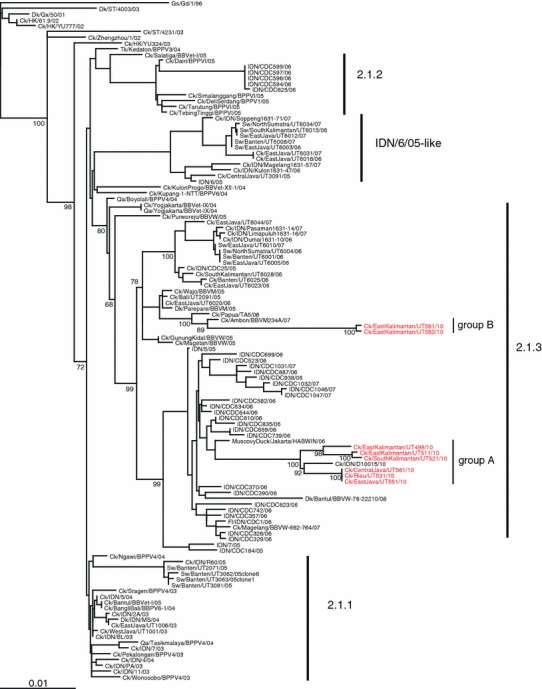

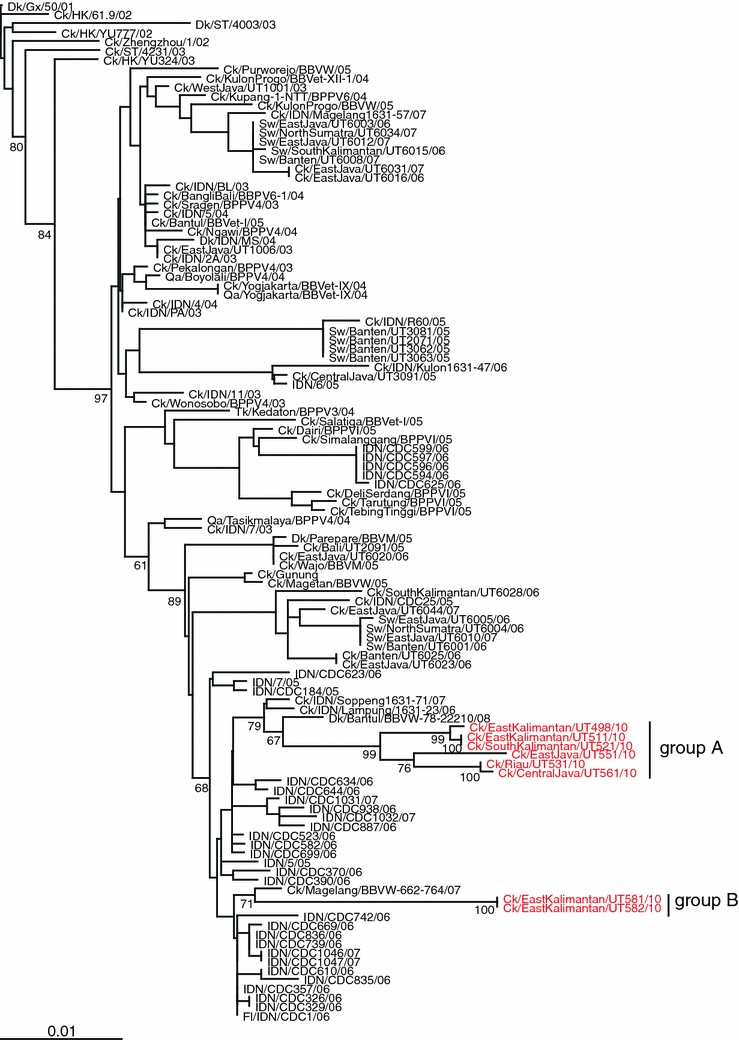
Phylogenetic relationships of the HA (**a**) and NA (**b**) genes of H5N1 influenza viruses in Indonesia. The numbers below or above the branch nodes indicate neighbor-joining bootstrap values. Analysis was based on nucleotides 77–1663 of the HA gene and 43–1037 of the NA gene. The HA and NA gene trees were rooted to A/goose/Guangdong/1/96 and A/duck/Guangxi/50/01, respectively. Colors indicate 2010 isolates (*red*). *Scale bar*, 0.01 nucleotide substitutions per site. *Ck* chicken; *Dk* duck; *Fl* feline; *Gd* Guangdong; *Gs* goose; *Gx* Guangxi; *HK* Hong Kong; *IDN* Indonesia; *ST* Shantou; *Sw* swine; *Tk* turkey; *Qa* quail

To characterize these eight chicken isolates, we sequenced their HA and NA genes and compared the sequences with those available in the GenBank database. Phylogenic analysis of the HA genes showed that they all belong to clade 2.1.3, based on the WHO/OIE classification, and composed two distinct clusters (Fig. 2a). Phylogenetic analysis of the NA genes showed similar phylogenetic relationships to those of the HA genes (Fig. 2b). Sequence comparison of the HA and NA genes of these eight isolates revealed that they could be divided into two groups: group A = Ck/EastKalimantan/UT498/10, Ck/EastKalimantan/UT511/10, Ck/SouthKalimantan/UT521/10, Ck/Riau/UT531/10, Ck/EastJava/UT551/10, and Ck/CentralJava/UT561/10; and group B = Ck/EastKalimantan/UT581/10 and Ck/EastKalimantan/UT582/10 (Table 2; Fig. 2a, b). For the HA genes, group A viruses had more than 85 nucleotide differences, resulting in 25 amino acid differences compared to group B viruses. Comparison of the NA genes showed that group A viruses differed by about 50 nucleotides compared to group B viruses, resulting in 18 amino acid differences.

**Table 2:** Summary of virus strains sequenced in this study

Group	Viruses	Clade (HA)	Collection	Sampling Province	Virus with highest % identity	
					HA	NA
A	Ck/EastKalimantan/UT498/10	2.1.3	April	East Kalimantan	Ck/IDN/D10015/10 (99%)	Dk/Bantul/BBVW-78-22210/08 (98%)
	Ck/EastKalimantan/UT511/10	2.1.3	April	East Kalimantan	Ck/IDN/D10015/10 (99%)	Dk/Bantul/BBVW-78-22210/08 (98%)
	Ck/SouthKalimantan/UT521/10	2.1.3	July	South Kalimantan	Ck/IDN/D10015/10 (99%)	Dk/Bantul/BBVW-78-22210/08 (98%)
	Ck/Riau/UT531/10	2.1.3	July	Riau (Sumatra)	Ck/IDN/D10015/10 (99%)	Dk/Bantul/BBVW-78-22210/08 (98%)
	Ck/EastJava/UT551/10	2.1.3	May	East Java	Ck/IDN/D10015/10 (99%)	Dk/Bantul/BBVW-78-22210/08 (98%)
	Ck/CentralJava/UT561/10	2.1.3	August	Central Java	Ck/IDN/D10015/10 (99%)	Dk/Bantul/BBVW-78-22210/08 (98%)
B	Ck/EastKalimantan/UT581/10	2.1.3	June	East Kalimantan	Ck/Ambon/BBVM234A/07 (97%)	Ck/Magelang/BBVW-662-764/07 (98%)
	Ck/EastKalimantan/UT582/10	2.1.3	July	East Kalimantan	Ck/Ambon/BBVM234A/07 (97%)	Ck/Magelang/BBVW-662-764/07 (98%)

A blast search of each HA nucleotide indicated that the virus with the highest identity with group A viruses was a chicken virus isolated in Indonesia in 2010 (A/chicken/Indonesia/D10015/10) with 99% identities. For group B viruses, a chicken virus isolate in Indonesia in 2007 (A/chicken/Ambon/BBVM234A/07) was genetically the closest strain with 97% homology. For the NA genes, the viruses with the highest identity were A/duck/Bantul/BBVW-78-22210/08 for group A viruses, and A/chicken/Magelang/BBVW-662-764/07 for group B viruses with 98% identities. The fact that the genetically closest viruses were all Indonesian isolates suggests that the eight isolates in this study have been evolving within Indonesia.

Some of the group A and B viruses were isolated from samples collected in Kalimantan province between April and July (Table 2), indicating that genetically distinct viruses circulated in the same area. On the other hand, two viruses from group A (A/chicken/Riau/UT531/10 and A/chicken/CentralJava/UT561/10) were isolated from samples collected in different provinces: Sumatra and Central Java, yet the HAs and NAs of these viruses were identical or identical except for one nucleotide, respectively. This finding suggests that these viruses may have been transferred from province to province, such as by human transport of infected chickens or via wild migratory birds.

Analysis of the HA amino acid sequences revealed that none of the isolates acquired any mutations that are known to be responsible for human-type receptor recognition. This finding suggests that unlike some H5N1 viruses isolated from chickens in Egypt [6], these Indonesian viruses are unable to recognize human-type receptors. No additions or lack of glycosylation sites were observed when we compared the HA sequences of the eight viruses with those of previous chicken isolates in Indonesia. Analysis of the NA amino acids showed that these eight isolates have the 20-amino acid stalk deletion that is associated with adaptation of influenza viruses from aquatic birds to territory poultry [7–10]. These eight isolates do not possess any of the amino acids in the catalytic and framework sites that are responsible for resistance to NA-inhibitor drugs [11–14]. Group A viruses did have a unique potential glycosylation site at amino acid position 50, which has also been observed in some H5N1 viruses isolated in China during 2001–2003, according to the sequence data in the influenza databases. The significance of this site is not yet known.

In summary, here, we isolated eight H5N1 influenza viruses from chickens and found that they all belong to clade 2.1.3, based on the WHO/OIE classification. These eight viruses were divided into two groups on the basis of nucleotide differences. We found that viruses that belong to the two groups co-circulated in the same province during the same season in Indonesia and that genetically almost identical viruses were circulating in different areas, suggesting that genetically distinct H5N1 viruses have been evolving and co-circulating in Indonesian countries.

To better understand the prevalence and adaptation of H5N1 influenza viruses in Indonesia, it is essential to perform comprehensive surveillance and to share the information obtained.
